# Binding of Phage-Encoded FlaGrab to Motile *Campylobacter jejuni* Flagella Inhibits Growth, Downregulates Energy Metabolism, and Requires Specific Flagellar Glycans

**DOI:** 10.3389/fmicb.2020.00397

**Published:** 2020-03-20

**Authors:** Jessica C. Sacher, Asif Shajahan, James Butcher, Robert T. Patry, Annika Flint, David R. Hendrixson, Alain Stintzi, Parastoo Azadi, Christine M. Szymanski

**Affiliations:** ^1^Department of Biological Sciences, University of Alberta, Edmonton, AB, Canada; ^2^Complex Carbohydrate Research Center, University of Georgia, Athens, GA, United States; ^3^Ottawa Institute of Systems Biology, University of Ottawa, Ottawa, ON, Canada; ^4^Department of Microbiology, University of Georgia, Athens, GA, United States; ^5^Department of Microbiology, University of Texas Southwestern Medical Center, Dallas, TX, United States

**Keywords:** bacteriophages, *Campylobacter jejuni*, flagella, protein glycosylation, pseudaminic acid, mass spectrometry, motility, bacterial surface structures

## Abstract

Many bacterial pathogens display glycosylated surface structures that contribute to virulence, and targeting these structures is a viable strategy for pathogen control. The foodborne pathogen *Campylobacter jejuni* expresses a vast diversity of flagellar glycans, and flagellar glycosylation is essential for its virulence. Little is known about why *C. jejuni* encodes such a diverse set of flagellar glycans, but it has been hypothesized that evolutionary pressure from bacteriophages (phages) may have contributed to this diversity. However, interactions between *Campylobacter* phages and host flagellar glycans have not been characterized in detail. Previously, we observed that Gp047 (now renamed FlaGrab), a conserved *Campylobacter* phage protein, binds to *C. jejuni* flagella displaying the nine-carbon monosaccharide 7-acetamidino-pseudaminic acid, and that this binding partially inhibits cell growth. However, the mechanism of this growth inhibition, as well as how *C. jejuni* might resist this activity, are not well-understood. Here we use RNA-Seq to show that FlaGrab exposure leads *C. jejuni* 11168 cells to downregulate expression of energy metabolism genes, and that FlaGrab-induced growth inhibition is dependent on motile flagella. Our results are consistent with a model whereby FlaGrab binding transmits a signal through flagella that leads to retarded cell growth. To evaluate mechanisms of FlaGrab resistance in *C. jejuni*, we characterized the flagellar glycans and flagellar glycosylation loci of two *C. jejuni* strains naturally resistant to FlaGrab binding. Our results point toward flagellar glycan diversity as the mechanism of resistance to FlaGrab. Overall, we have further characterized the interaction between this phage-encoded flagellar glycan-binding protein and *C. jejuni*, both in terms of mechanism of action and mechanism of resistance. Our results suggest that *C. jejuni* encodes as-yet unidentified mechanisms for generating flagellar glycan diversity, and point to phage proteins as exciting lenses through which to study bacterial surface glycans.

## Introduction

Targeting bacterial virulence without threatening essential cellular processes is a strategy that can help minimize antimicrobial resistance (Heras et al., 2015). Many bacterial pathogens display glycosylated surface structures that promote virulence in human and animal hosts, making these structures attractive drug targets (Szymanski and Wren, 2005). However, understanding the role and diversity of bacterial surface glycans has lagged behind other fields of biology, due in part to a lack of a predictable code for their synthesis, difficulties in characterizing the detected glycans, and a lack of appropriate reagents with specificity for bacterial glycans, which are much more diverse at the monosaccharide level than those found in eukaryotes.

*Campylobacter jejuni* causes severe diarrheal disease around the world (Bolton, 2015) and has been associated with human growth stunting in low-resource settings (Amour et al., 2016). *C. jejuni* relies on glycosylated flagella to colonize the gastrointestinal epithelium of humans and animals (Guerry, 2007). Its flagellin proteins (FlaA and FlaB) are among the most heavily glycosylated bacterial proteins known (Logan, 2006), with up to 10% of their molecular weight due to glycans (Guerry and Szymanski, 2008). Unlike some other flagellated bacterial pathogens, such as *Pseudomonas aeruginosa*, whose flagella assemble with or without glycosylation (Schirm et al., 2004), flagellar glycosylation is essential to *C. jejuni* flagellar filament biogenesis, and is thus essential for its motility and virulence (Goon et al., 2003; Logan, 2006). The importance of glycosylated flagella to *C. jejuni* is further illustrated by the fact that it devotes approximately 3% of its small, 1.6-Mb genome to genes associated with flagellar glycosylation, and that two of its three sigma factors are devoted to regulating flagellar biosynthesis and glycosylation (Logan, 2006). Given the importance of flagellar glycosylation to *C. jejuni* virulence and the high proportion of genetic resources it devotes to this process, understanding *C. jejuni* flagellar glycosylation has a high likelihood of generating key insights toward better controlling this pathogen.

*Campylobacter* species glycosylate their flagella with two major nonulosonic acids (nine-carbon sugars): pseudaminic acid (5,7-diacetamido-3,5,7,9-tetradeoxy-L-*glycero*-α-L-*manno*-nonulosonic acid, also known as Pse5Ac7Ac or Pse) and legionaminic acid (5,7-diamino-3,5,7,9-tetradeoxy-D-*glycero*-D-*galacto*-nonulosonic acid, also known as Leg5Ac7Ac or Leg), both of which have a molecular mass of ~316 Da. In addition, several derivatives of these glycans have been identified on *C. jejuni* flagella to date. The genetic and biochemical pathways for biosynthesis of many of these glycans have been studied extensively in *C. jejuni* and *Campylobacter coli;* this has primarily been done by analyzing trypsin-digested flagella by mass spectrometry (MS) and by analyzing nucleotide-activated flagellar glycan precursors in the cytoplasm of defined mutants using MS and nuclear magnetic resonance (NMR) spectroscopy (Guerry et al., 1990, 1991, 2006; Thibault et al., 2001; Logan et al., 2002; Goon et al., 2003; Schirm et al., 2003, 2005; Szymanski et al., 2003; McNally et al., 2006, 2007; Schoenhofen et al., 2006; Guerry, 2007; Ewing et al., 2009; Howard et al., 2009). However, these methods are technically demanding, expensive, and time-intensive, and thus the already impressive repertoire of *Campylobacter* flagellar glycans has come from the analysis of only a few strains. Furthermore, linking genotype with phenotype in this organism, especially in the context of flagellar biogenesis and modification, is complicated by the presence of phase-variable polynucleotide tracts in genes encoding glycosylation enzymes. This leads to frequent on/off switching of genes (~1/1,000 generations) due to slipped-strand mispairing during DNA replication (Lango-Scholey et al., 2016). For example, *C. jejuni* 11168, the first genome-sequenced strain of the species, harbors poly-deoxyguanosine (poly-G) tracts in at least 28 of its genes (Parkhill et al., 2000; Lango-Scholey et al., 2016), 10 of which are found within its ~50-gene flagellar glycosylation locus (Hitchen et al., 2010). Given these challenges, much of the diversity in *Campylobacter* flagellar glycans, as well as the mechanisms that drive this diversity, remain unknown.

Bacteriophages (phages), viruses specific for bacteria, commonly target bacterial surface structures, many of which are glycosylated (Simpson et al., 2015). It follows that evolutionary pressure from phages has likely contributed to driving the diversity in bacterial surface glycans. *Campylobacter* phages are known to recognize host cells by binding to glycosylated surface structures, such as flagella (Baldvinsson et al., 2014) and capsular polysaccharide (CPS) (Sørensen et al., 2011; Gencay et al., 2018); however, these interactions have not been characterized in detail. Previously, we observed that Gp047 (now renamed FlaGrab), a conserved *Campylobacter* phage protein (Javed et al., 2014), binds to *C. jejuni* flagella displaying the nine-carbon monosaccharide 7-acetamidino-pseudaminic acid (Pse5Ac7Am) (Javed et al., 2015a), and that this binding inhibits cell growth (Javed et al., 2015b). However, the mechanism of this growth inhibition, as well as how *C. jejuni* might resist this activity, are not well-understood.

The aim of this study was to better characterize the interaction between phage-encoded FlaGrab and *C. jejuni* cells from two perspectives: (1) how FlaGrab exerts its growth inhibitory activity, and (2) how cells are able to resist this activity. We hypothesized that FlaGrab binding to *C. jejuni* flagella would create increased drag that would signal cells to alter gene expression in a way that would slow cell growth. To test this, we used RNA-Seq to observe changes in gene expression in response to FlaGrab, and examined the role of flagellar motility in FlaGrab activity. We hypothesized that *C. jejuni* might resist FlaGrab activity by displaying flagellar glycans different from those expressed by FlaGrab-susceptible strains. To test this, we used transmission electron microscopy and immunogold labeling to analyze whether *C. jejuni* strains resistant to FlaGrab activity evaded binding by the protein. To understand the mechanism by which strains were able to evade binding, we examined the flagellar glycosylation genetic loci and used mass spectrometry to characterize the flagellar glycans of FlaGrab-resistant strains. Our results show that FlaGrab binding to motile *C. jejuni* flagella leads to changes in expression of energy metabolism genes, and that cells resistant to this binding display altered flagellar glycans.

## Materials and Methods

### Bacterial Growth Conditions

*C. jejuni* strains were grown on NZCYM (Difco) agar, supplemented with 50 μg/mL kanamycin where needed, at 37°C under microaerobic conditions (85% N_2_, 10% CO_2_, 5% O_2_). *E. coli* was grown on LB agar supplemented with 50 μg/mL kanamycin where needed. The list and sources of bacterial strains used in this study are given in Table 1.

**Table 1:** List of bacterial strains and plasmids used in this study.

**Name**	**Source**	**References**
*C. jejuni* NCTC 11168 (MP21)	Human enteropathy	Gundogdu et al., 2007
*C. jejuni* NCTC 12567	Chicken	McNally et al., 2007
*C. jejuni* NCTC 12660	Chicken	Frost et al., 1999
*C. jejuni* NCTC 12661	Pigeon	Frost et al., 1999
*C. jejuni* NCTC 12664	Chicken	Frost et al., 1999
*C. jejuni* NCTC 11168 Δ*cj1295*	*C. jejuni* NCTC 11168 (MP21)	This work
*C. jejuni* 11168 Δ*motA*	*C. jejuni* 11168 (MP21)	This work
*C. jejuni* 11168 Δ*motB*	*C. jejuni* 11168 (MP21)	This work
pGEX_*ccgp047*	Expression construct of GST-fused CC-Gp047 (CC-FlaGrab) in pGEX 6P-2	Javed et al., 2013
pGEX_*ncgp047*	Expression construct of GST-fused NC-Gp047 (NC-FlaGrab) in pGEX 6P-2	Javed et al., 2013
pDRH3330 (pUC19::*motA*-cat-*rpsL*)	*motA* mutagenesis construct	Beeby et al., 2016
pDRH3331 (pUC19::*motB*-cat-*rpsL*)	*motB* mutagenesis construct	Beeby et al., 2016

### Expression and Purification of FlaGrab and Its Derivatives

FlaGrab was expressed as an N-terminal glutathione-S-transferase (GST) fusion protein as described previously (Kropinski et al., 2011). CC-FlaGrab [the C-terminal quarter of FlaGrab, previously shown to harbor the growth inhibitory activity (Javed et al., 2015b)] and NC-FlaGrab [the N-terminal quarter of FlaGrab, previously shown to retain no growth inhibitory activity (Javed et al., 2015b)] were expressed in *E. coli* BL21 as GST-fused proteins and purified as described previously (Javed et al., 2013), with the exception that proteins were eluted in 10 mM reduced L-glutathione, 50 mM HEPES, and 1 mM DTT at pH 9.0. The list of plasmids used in this study are listed in Table 1.

### Total RNA Extraction

Cells were harvested from overnight NZCYM plate cultures, pelleted and washed once in NZCYM broth and set to an OD_600_ of 0.05 (2 × 10^8^ CFU/mL) in 20 mL NZCYM broth in 125-mL Erlenmeyer flasks. Cells were grown under microaerobic conditions with agitation at 200 rpm. After 4.5 h incubation, CC-FlaGrab was added to a final concentration of 25 μg/mL. As negative controls, 6 mL HEPES buffer or 6 mL NC-FlaGrab were used. Flasks were incubated 30 min before the entire contents of each was harvested and the RNA stabilized using 0.1 volumes of ice cold 10% buffered phenol in 100% ethanol (Palyada et al., 2004). Total RNA was extracted from each sample using a hot phenol method as previously described (Palyada et al., 2004). Genomic DNA was removed from the samples using RNAse-free DNAse I (Epicenter) (37°C for 30 min) and cleaned using the Zymo RNA Clean & Concentrator. PCR was used to confirm the absence of residual DNA and the DNase treatment was repeated until the absence of genomic DNA was confirmed. Total RNA quality was assessed using an Agilent Bioanalyzer and RNA was stored at −80°C until further use. This experiment was done in biological triplicate, with the exception of the NC-FlaGrab control, which was done only once.

### RNA Sequencing

Samples were subsequently depleted of rRNA using the RiboZero Bacterial kit according to the manufacturer's instructions and rRNA depletion was confirmed using the Agilent Bioanalyzer RNA 6000 Pico Kit. The Ion Total RNA-seq kit was used to construct strand-specific barcoded sequencing libraries according to the manufacturer's instructions. Following library construction, each library was quality-checked and quantified using the Bioanalyzer High Sensitivity DNA kit. Once all libraries were completed, they were pooled together in equimolar amounts and were templated using the Ion PI Hi-Q kit. The templated beads were sequenced on an Ion Torrent Proton using the Ion PI Hi-Q sequencing 200 kit on a single Proton V2 chip.

The raw sequencing reads were demultiplexed by the Ion Torrent suite software and mapped to the 11168 genome using STAR (Love et al., 2014). The raw demultiplexed sequencing reads have been deposited at the NCBI SRA archive under accession number PRJNA580017. Reads aligning to coding regions were counted using HT-seq using the default settings (Dobin et al., 2013). DESeq2 was used to identify differentially expressed transcripts between the control and CC-FlaGrab treated cells. Genes with a false discovery rate (FDR)-corrected *p* < 0.05 were considered differentially expressed. We also conducted gene set enrichment analysis (GSEA) on *C. jejuni* Kyoto Encyclopedia of Genes and Genomes (KEGG) pathways using the generally applicable gene set enrichment (GAGE) method (Luo et al., 2009) with a minimum FDR cutoff of <0.1 (Love et al., 2014).

### Generation of *motA, motB*, and *cj1295* Mutants

To generate *motA* and *motB* mutants, *C. jejuni* 11168 was transformed by natural transformation with the pDRH3330 (*motA*) or pDRH3331 (*motB*) mutation constructs published by Beeby et al. (2016). To generate a *cj1295* mutant, *C. jejuni* 11168 was naturally transformed with the *cj1295* mutation construct (p1295) described by Hitchen et al. (2010).

For natural transformation of mutant constructs, an overnight culture of *C. jejuni* cells was streaked onto BHI containing 2% yeast extract (BHIY), grown for 6 h under the conditions described above, and harvested into PBS. Cells were washed once in PBS, resuspended in 250 μL PBS, spotted (20–30 × 10-μL spots) onto pre-warmed (37°C) BHIY plates and allowed to absorb into agar ~5 min. p1295 DNA (20 μg/mL in water) was spotted (~10-μL spots) atop each spot of cell suspension and allowed to dry ~5 min. Plates were incubated for 12–16 h, then the contents of the plates were harvested and spread or streaked across 4–6 BHI plates containing 50 μg/mL kanamycin. Plates were incubated until colonies appeared (3–5 days). Isolated colonies were patched onto kanamycin-containing BHI plates and grown overnight prior to confirmation of successful mutagenesis by colony PCR (OneTaq, NEB) using gene-specific primers (Hitchen et al., 2010; Beeby et al., 2016). This protocol has been deposited into Protocols.io (dx.doi.org/10.17504/protocols.io.magc2bw).

### Immunogold Labeling and Transmission Electron Microscopy

Immunogold labeling and transmission electron microscopy was done as described previously (Javed et al., 2015a), with some exceptions. Briefly, *C. jejuni* cells (OD_600_ = 3.0) were harvested from a plate of overnight growth in NZCYM broth, incubated with Formvar-coated copper grids for 45 min, washed 1 × 3 min with PBS, then fixed by incubating in 2.5% paraformaldehyde in PBS for 20 min. Grids were then washed 3 × 3 min with PBS, incubated in blocking solution (PBS containing 5% bovine serum albumin and 0.05% Tween) for 35 min, then in freshly purified GST-tagged FlaGrab (0.2 mg/mL diluted 1:25 in blocking solution) for 45 min. Grids were then washed 3 × 3 min in blocking solution, incubated in a rabbit anti-FlaGrab antibody solution (diluted 1:50 in blocking solution) for 45 min, washed 3 × 3 min as above, and incubated in a solution of goat anti-rabbit IgG (whole molecule) conjugated to 10-nm gold particles (Sigma, diluted 1:50 in blocking solution) for 45 min. Finally, grids were sequentially washed (3 × 3 min each) with blocking solution, PBS, and distilled water, then air-dried on Whatman filter paper overnight. All steps were done at room temperature. Grids were examined using a transmission electron microscope (JEOL JEM1011; JEOL, Inc., Peabody, MA, USA). Images were captured using a high contrast 2k × 2k mid-mount digital camera (Advanced Microscopy Techniques, Corp., Woburn, MA, USA). This protocol has been deposited into Protocols.io (dx.doi.org/10.17504/protocols.io.mv5c686).

### FlaGrab Growth Clearance Assay

Bacterial growth clearance was tested by spotting phage protein onto a freshly inoculated bacterial suspension using the overlay agar method described previously, with some modifications (Javed et al., 2015b). Briefly, overnight bacterial cultures were harvested in NZCYM broth and set to an OD_600_ of 0.35. A 5-mL aliquot of this suspension was transferred to a standard sized empty Petri dish and incubated at 37°C without shaking for 4 h under microaerobic conditions. The suspension was then set to an OD_600_ of 0.5, and 200 μL of this was mixed with 5 mL sterile 0.6% molten NZCYM agar at 45°C. This suspension was poured onto the surface of a pre-warmed (37°C) NZCYM plate containing 1.5% agar. Plates were allowed to solidify for 15 min and then 10 μL UV-sterilized FlaGrab or CC-FlaGrab solution (0.91–1.3 mg/mL in 10 mM reduced L-glutathione, 50 mM Tris, 1 mM DTT, pH 9.0) was spotted onto the agar surface and allowed to completely soak into the agar before inverting the plate and incubating at 37°C under microaerobic conditions. Zones of growth clearance were observed after 18–24 h.

### Sequence Alignments and Analysis

Nucleic acid sequence information for *cj1293*-*cj1342* (the flagellar glycosylation locus in 11168) for strains 11168, 12567, 12660, 12661, and 12664 was obtained from NCBI (Gundogdu et al., 2007; Sacher et al., 2018a,b). Nucleotide and amino acid sequence alignments and phylogenetic trees were generated using Geneious version 8 [http://www.geneious.com (Kearse et al., 2012)]. Alignment figures were generated using T-Coffee (Di Tommaso et al., 2011) and Boxshade (http://sourceforge.net/projects/boxshade/). Black shading indicates the consensus sequence, gray shading indicates similar residues.

### Preparation of Cell-Free Flagella for MS

Cell-free flagella were prepared for MS as described previously with some modifications (Javed et al., 2015a). Briefly, bacterial growth was harvested from 20 to 40 NZCYM agar plates, washed with PBS and resuspended in 100–150 mL PBS and stored up to 7 days at −80°C. Cells were thawed by vortexing and flagella were sheared from cells using a Polytron homogenizer for 6 × 30 s (allowing 30 s incubation on ice between rounds) at max rpm on ice. Cells were then removed by centrifugation at 8,700 × g for 20 min at 4°C. To collect flagellar filaments, the resulting supernatant was ultracentrifuged at 207,870 × g, 4°C for 1.5 h using 6 × 30-mL tubes (Beckman). Pellets were washed 3 times by resuspending each pellet in 20 mL distilled water and ultracentrifuging again as above, giving a total of 4 × 1.5 h ultracentrifuge runs. After washing, pellets were resuspended and pooled into a total of 1 ml distilled water and incubated at −20°C until use.

### Flagellin Protease Digestion

First, 100 μg of flagella protein sample was dissolved in 25 μL of digestion buffer [50 mM aqueous ammonium bicarbonate (NH_4_HCO_3_) buffer]. Then, 25 μL of dithiothreitol (DTT) solution (25 mM) was added to the samples and incubated at 45°C for 45 min. Subsequently, carbamidomethylation was performed by adding 25 μL of iodoacetamide (IAA) solution (90 mM) and incubating at room temperature for 45 min in the dark. The sample was then dialyzed against double distilled water and lyophilized. Flagellin was digested by adding 12.5 μL sequencing-grade trypsin (Promega, 0.4 μg/μL) and incubating at 37°C for 12 h. The digest was desalted by C18 solid phase extraction cartridges and then dried under a speed vacuum. The peptides and glycopeptides were subsequently re-dissolved in 0.1% formic acid in water and stored at −30°C until analysis by nano-LC-MS/MS.

### Nano-LC-MS/MS Acquisition

Desalted flagellin digests were analyzed on an Orbitrap Fusion Tribrid mass spectrometer (Thermo Scientific) equipped with a nanospray ion source and connected to a Dionex binary solvent system. Pre-packed nano-LC columns (15 cm in length, 75 μm in internal diameter) filled with 3 μm C18 material were used for chromatographic separation of glycoprotein digests. Precursor ion scanning was performed at 120,000 resolution in an Orbitrap analyzer, and precursors at a time frame of 3 s were selected for subsequent fragmentation using higher energy collision dissociation (HCD) at a normalized collision energy of 28 and collision-induced dissociation (CID) at an energy of 35. The threshold for triggering an MS/MS event was set to 500 counts. The fragment ions were analyzed on an Orbitrap after HCD and CID fragmentation at a resolution of 30,000. Charge state screening was enabled, and precursors with unknown charge state or a charge state of +1 were excluded (positive ion mode). Dynamic exclusion was enabled with an exclusion duration of 10 sec.

### MS Data Analysis

The LC-MS/MS spectra of the tryptic digest of proteins were searched against the FlaA and FlaB protein sequence of the respective strains in FASTA format using Byonic 2.3 software with trypsin as the digestion enzyme. Carbamidomethylation of cysteine and oxidation of methionine were selected as variable modifications. Glycan modifications such as Pse5Ac7Ac (m/z = 316.12705), Pse5Ac7Am (m/z = 315.14304), DMGA-Pse5Ac7Ac (m/z = 390.16383), and DMGA-Pse5Ac7Am (m/z = 389.17981) were used as variable modifications on serines/threonines. The LC-MS/MS spectra were analyzed manually for the glycopeptide fragmentation on HCD and CID with the support of Xcalibur software. To identify glycans, the spectra of the glycopeptides were evaluated for glycan neutral loss patterns, oxonium ions, and glycopeptide fragmentations. Unknown derivatives of pseudaminic acid were identified based on the presence of oxonium ions and neutral losses on the spectra of glycopeptides bearing glycosylation sites.

## Results

### FlaGrab-Treated Cells Upregulate Membrane Proteins and Downregulate TCA Cycle Genes

To determine the mechanism of FlaGrab growth inhibition of *C. jejuni* 11168 cells, we sequenced total mRNA (RNA-seq) from cells incubated with CC-FlaGrab [the C-terminal quarter of FlaGrab, previously shown to possess the growth inhibitory activity (Javed et al., 2015b)] for 30 min and compared their transcriptomic profiles with cells incubated with buffer. As an additional negative control, we extracted RNA from buffer-exposed cells and from cells exposed to NC-FlaGrab (the N-terminal quarter of FlaGrab, previously shown to retain no growth inhibitory activity). PCA plot analysis of all samples showed that the NC-FlaGrab clustered with the buffer controls, suggesting that FlaGrab only impacts gene expression when capable of binding to host targets (Figure S1).

**Figure 1:**
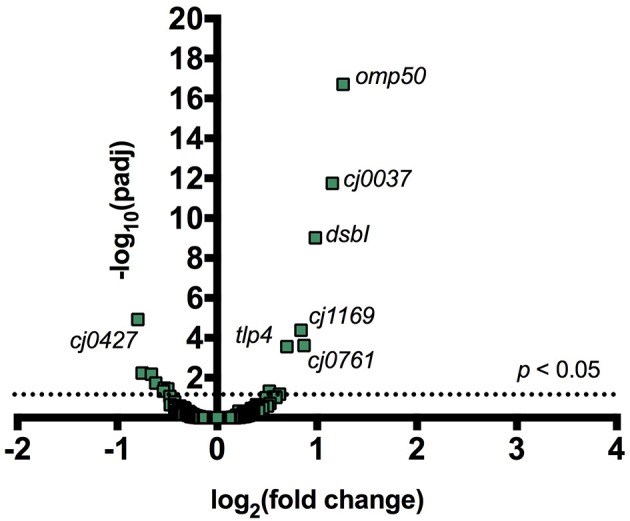
Addition of CC-FlaGrab to *C. jejuni* 11168 cells leads to changes in gene expression. For all transcribed *C. jejuni* 11168 genes, the negative log of the FDR-adjusted *p-*value is plotted against the log_2_ fold change in transcription following CC-FlaGrab treatment compared with buffer-treated controls.

Incubating *C. jejuni* 11168 with CC-FlaGrab altered the expression of genes encoding energy metabolism enzymes, membrane proteins, and periplasmic proteins (Figure 1 and Table 2). Upregulated genes include *omp50*, which encodes a major *C. jejuni* porin and phosphotyrosine kinase also known as Cjtk (Corcionivoschi et al., 2012). Other upregulated genes include *cj1169c* (a periplasmic protein-encoding gene upstream of *omp50*); *cj0037c* (cccC), which encodes cytochrome C (Guccione et al., 2017); *dsbI*, which encodes a disulfide bond forming protein; *tlp4*, which encodes a chemotaxis receptor; *metB*, which encodes a methionine biosynthesis enzyme; and *cj0761*, which encodes a hypothetical protein. Downregulated genes include *sdhAB* (*mrfAB*), which are misannotated as succinate dehydrogenases but are likely to encode methylmenaquinol:fumarate reductases, which function in the tricarboxylic acid (TCA) cycle (Weingarten et al., 2009; Kassem et al., 2014; Guccione et al., 2017). Other downregulated genes include *cj0426*, which encodes an ABC transporter ATP binding protein, the downstream gene *cj0427, rpmF*, which encodes a 50s ribosomal protein, and *cj0911* and *cj1485c*, which encode hypothetical periplasmic proteins. KEGG pathway analysis showed that several categories were enriched in the downregulated genes upon CC-FlaGrab exposure, including the TCA cycle, carbon metabolism, and oxidative phosphorylation pathways (Table 3).

**Table 2:** Differentially expressed *C. jejuni* 11168 genes upon CC-FlaGrab treatment.

**Gene**	**Annotation**	**Fold change**	***p-*value^a^**
*omp50 (cjtk)*	50 kDa outer membrane protein precursor, phosphotyrosine kinase^b^	2.39	<0.01
*cj0037c (cccC)*	Putative cytochrome C	2.22	<0.01
*dsbI*	Disulphide bond formation protein	1.98	<0.01
*cj0761*	Hypothetical protein Cj0761	1.83	<0.01
*cj1169c*	Putative periplasmic protein	1.79	<0.01
*tlp4*	Putative methyl-accepting chemotaxis signal transduction protein	1.62	<0.01
*metB*	Putative *O*-acetylhomoserine (thiol)-lyase	1.44	0.05
*cj0426*	Putative ABC transporter ATP-binding protein	−1.41	0.04
*cj0911*	Putative periplasmic protein	−1.44	0.03
*sdhA (mrfA)*	Possible methylmenaquinol:fumarate reductase (TCA cycle)^c^	−1.45	0.05
*sdhB (mrfB)*	Possible methylmenaquinol:fumarate reductase (TCA cycle)^c^	−1.53	0.02
*rpmF*	50S ribosomal protein L32	−1.58	0.01
*cj1485c*	Putative periplasmic protein	−1.68	0.01
*cj0427*	Hypothetical protein Cj0427	−1.74	<0.01

a *For each gene, fold change in expression post CC-FlaGrab treatment is listed followed by the FDR-adjusted p-value in brackets*.

b *Genbank annotation updated according to Corcionivoschi et al. (2012)*.

c *Genbank annotation updated according to Kassem et al. (2014)*.

**Table 3:** Statistically significant downregulated KEGG pathways following *C. jejuni* 11168 exposure to CC-FlaGrab.

**Significantly downregulated KEGG pathways^a^**	***p-*value**
cje00020 Citrate cycle (TCA cycle)	0.001
cje01200 Carbon metabolism	0.006
cje00190 Oxidative phosphorylation	0.019
cje03010 Ribosome	0.019
cje01130 Biosynthesis of antibiotics	0.019
cje01120 Microbial metabolism in diverse environments	0.019
cje00650 Butanoate metabolism	0.020

a *Differentially expressed host genes for each condition were subjected to gene set enrichment analysis (GSEA) on annotated Kyoto Encyclopedia of Genes and Genomes (KEGG) pathways using GAGE with an FDR cutoff of <0.1*.

### FlaGrab Binding to Flagellar Motor Mutants Δ*motA* and Δ*motB* Does Not Cause Growth Inhibition

**Figure 2:**
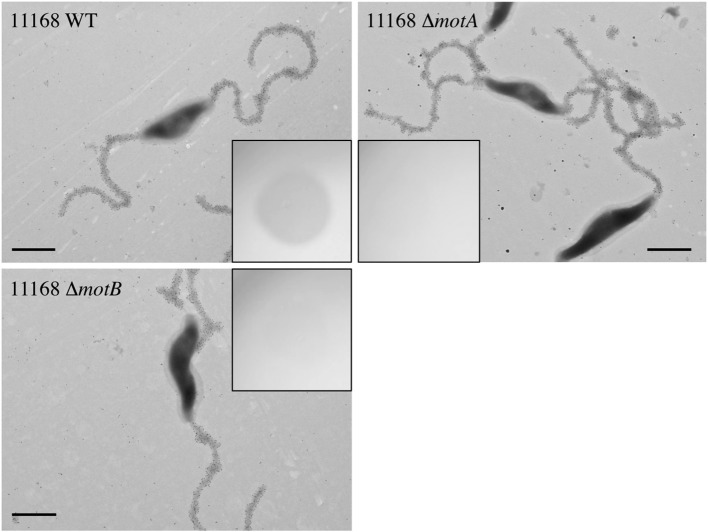
*C. jejuni* 11168 wild type, **Δ***motA* mutant, and **Δ***motB* mutant cells are similarly bound by CC-FlaGrab, as shown by immunogold labeling with FlaGrab-specific antibodies and transmission electron microscopy. **(Inset)** Only wild type cell growth is cleared by CC-FlaGrab, as shown by digital images of overnight growth on soft agar overlay plates following spotting with equal concentrations of CC-FlaGrab. All images are representative of experiments done in duplicate. Scale bars represent 800 nm.

We next sought to understand how FlaGrab binding to bacterial flagella might lead to the observed effects on cell physiology. The proton motive force is hypothesized to power *C. jejuni* flagellar rotation through the MotA and MotB stator proteins, as in many other motile bacteria (Blair and Berg, 1990; Hosking et al., 2006; Morimoto et al., 2010; Kojima et al., 2018). Since flagellar motility is reduced upon FlaGrab binding (Javed et al., 2015a), we hypothesized that the cell might sense increased FlaGrab-induced flagellar stiffness and increase proton flow through the flagellar motor channel to compensate for this inhibition of flagellar rotation. This increased proton flow might in turn disrupt the electron transport chain (as proposed by Flint et al., 2014), which could prompt cells to respond by altering transcription of energy metabolism pathways. We predicted that if this were true, flagellar motility mutants, such as **Δ***motA* and **Δ***motB*, which do not express functional flagellar motors, would resist FlaGrab-induced clearing (Beeby et al., 2016; Ribardo et al., 2019). To test this, we generated insertional mutants in each of **Δ***motA* and **Δ***motB* in *C. jejuni* 11168 and confirmed that these mutants were non-motile (Figure S2). We then tested their binding and susceptibility to FlaGrab. We used immunogold labeling and transmission electron microscopy to show that binding to these mutants occurred to wild type levels (Figure 2), but when we tested growth inhibition of the **Δ***motA* and **Δ***motB* mutants using a soft agar spot assay, we found that FlaGrab was unable to clear the growth of these mutants (Figure 2, inset). This result supported our hypothesis that flagellar function is required for FlaGrab-mediated growth inhibition.

### Some *C. jejuni* Strains Display Reduced Susceptibility to FlaGrab-Induced Growth Inhibition

To better understand the mechanisms by which *C. jejuni* may resist FlaGrab growth inhibition, we sought to identify strains resistant to this activity. We identified four *C. jejuni* strains, 12567, 12660, 12661, and 12664, which were naturally reduced in their ability to be cleared by FlaGrab and CC-FlaGrab compared to 11168 (Javed et al., 2013) (Table 4). Strain 12567 was cleared to near-11168 levels, while strain 12664 was not cleared. Strain 12660 displayed reduced clearing, and strain 12661 was generally not cleared, although one biological replicate of this strain was cleared well. Motility of these strains did not correlate with susceptibility to FlaGrab-induced clearance; most strains displayed similar motility to 11168, with the exception of 12661, which was significantly less motile (49%, *p* = 0.008) than 11168 (Figure S3).

**Table 4:** Growth clearance of *C. jejuni* strains by FlaGrab and CC-FlaGrab.

**Strain**	**FlaGrab growth clearance^a^**		**CC-FlaGrab growth clearance^a^**	
*C. jejuni* NCTC 11168	+++	+++	++	+
*C. jejuni* 12567	+	++	+	-
*C. jejuni* NCTC 12660	+	+	+	-
*C. jejuni* NCTC 12661	-	-	++	-
*C. jejuni* NCTC 12664	-	-	-	-

a *Degree of growth clearance following spotting of full-length FlaGrab or CC-FlaGrab on growing C. jejuni cells and overnight incubation. Clearing was scored visually. (+++) = very clear, (++) = slightly clear, (+) = very faint clearing, (-) = no clearing. Each column represents a separate biological replicate*.

### *C. jejuni* Strains Resistant to FlaGrab-Induced Growth Inhibition Also Display Reduced FlaGrab Binding

**Figure 3:**
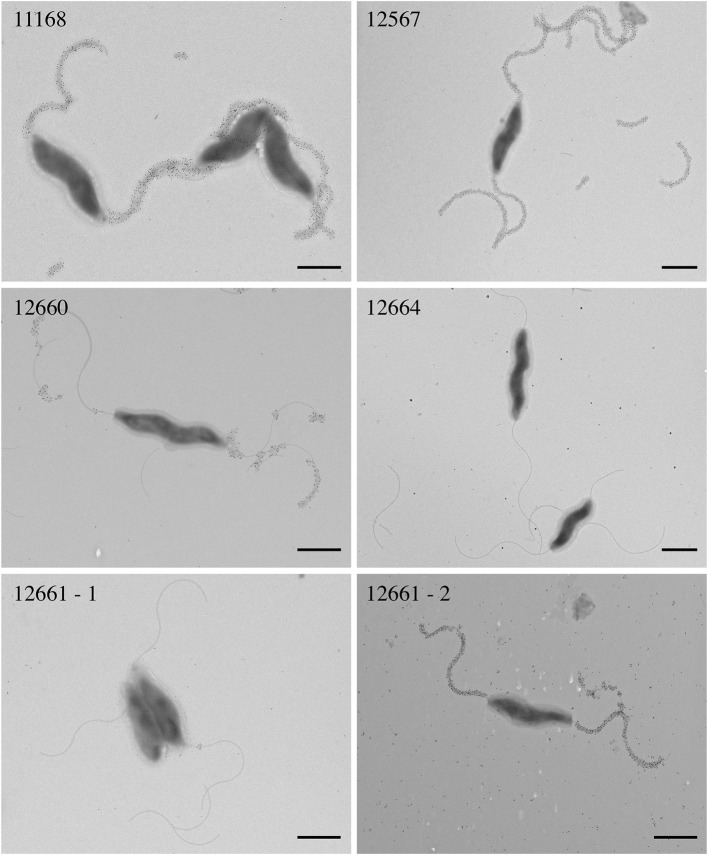
Transmission electron microscopy with immunogold labeling using CC-FlaGrab followed by anti-FlaGrab antibodies and then immunogold-conjugated secondary antibodies to probe *C. jejuni* strains. From top left: strains *C. jejuni* 11168, 12567, 12660, 12664, 12661-1, and 12661-2. Strain 12661 is depicted twice to show the two phenotypes that were observed with this strain. Scale bars represent 800 nm.

We hypothesized that reduced FlaGrab clearing would correlate with reduced binding. To test this, we probed cells with immunogold-labeled FlaGrab and examined them using transmission electron microscopy. We found that 12567 displayed robust FlaGrab binding (Figure 3), while 12660 displayed intermediate levels of binding. Interestingly, we found that biological replicates of 12661 showed either no binding or robust binding to FlaGrab (*n* = 2 each). Finally, we observed a complete lack of FlaGrab binding to 12664. Although neither the growth inhibition assay nor the immunogold-labeling assay provides quantitative results, the level of growth inhibition observed for these strains appeared to correlate with the levels of FlaGrab binding to the strains. These results suggested that reduced FlaGrab binding may explain the observed resistance to FlaGrab achieved by these strains.

### *C. jejuni* Strains Resistant to FlaGrab Binding Encode all Genes Required for Biosynthesis and Transfer of Pse5Ac7Am

We next sought to understand how cells achieved altered levels of FlaGrab binding. As we previously showed that FlaGrab requires Pse5Ac7Am for binding to *C. jejuni* 11168 flagella (Javed et al., 2015a), we hypothesized that strains displaying reduced levels or differing patterns of FlaGrab binding would express different levels of Pse5Ac7Am on their flagella. Pse5Ac7Ac is synthesized by the sequential action of PseB, PseC, PseH, PseG, PseI, and PseF (Logan, 2006). Pse5Ac7Am is formed by adding the Am group to Pse5Ac7Ac by PseA (Guerry et al., 2006). Pse5Ac7Ac and Pse5Ac7Am are then transferred to flagellin in the cytoplasm by the action of separate transferases: PseE transfers Pse5Ac7Ac, while PseD transfers Pse5Ac7Am (Karlyshev et al., 2002; Karlyshev and Wren, 2005; Guerry et al., 2006). *C. jejuni* and *C. coli* require Pse5Ac7Ac biosynthesis for flagellar biogenesis (Goon et al., 2003), and all strains examined here are flagellate, and thus, we predicted that all strains would encode the genes required for Pse5Ac7Ac synthesis and transfer onto Fla protein subunits. However, we hypothesized that 12664, which is unable to bind FlaGrab, would encode a non-functional version of a gene involved in Pse5Ac7Am biosynthesis and/or transfer (i.e., *pseA* and/or *pseD*). We hypothesized that 12661, which we found to variably bind FlaGrab, might express a phase-variable version of one or both of these genes. To test these hypotheses, we analyzed the sequenced genomes for these strains (Sacher et al., 2018a,b) and examined the region spanning *cj1293-cj1343*, which was previously established as the flagellar glycosylation locus in *C. jejuni* 11168 (Parkhill et al., 2000).

**Figure 4:**
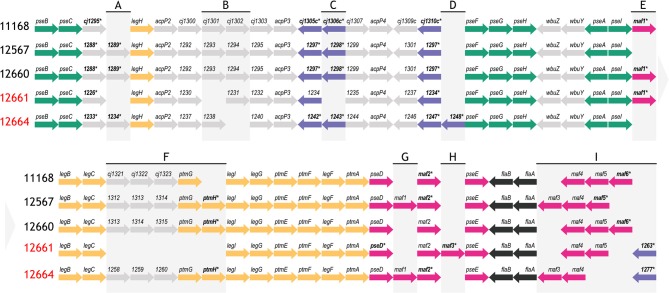
Schematic depicting the flagellar glycosylation loci of strains *C. jejuni* 12567, 12660, 12661 and 12664, as compared to *C. jejuni* 11168. Strain names indicated in red refer to strains that either variably (12661) or consistently (12664) showed a lack of FlaGrab binding by immunogold/TEM. Asterisks/bolded genes contain a phase-variable poly-G tract. Colors represent genes involved in the same biological pathway (green: *pse* biosynthesis, yellow: *leg* biosynthesis, black: flagellar filament biosynthesis) or genes within the same family according to predicted function (purple: DUF2920 domain-containing genes, pink: *maf* genes). Other genes are indicated in gray. **(A)–(I)** are used to indicate regions of heterogeneity in gene content between strains, which are referred to in the text. Arrow direction indicates direction of transcription. Gene lengths are not to scale. Pseudogenes are not depicted.

In terms of gene content, we found a high degree of similarity at the flagellar glycosylation loci of all five strains (Figure 4). Compared to 11168, which encodes 47 genes in this locus, we found that 12567, 12660, and 12664 had between 46–48 genes, while 12661 encodes only 40. This is in contrast to *C. jejuni* 81–176, another well-characterized strain, which encodes only 24 of the 47 flagellar glycosylation genes encoded by 11168 (Guerry et al., 2006). Notably, we found that similarly to 11168, all four strains (12567, 12660, 12661, and 12664) encoded copies of all genes required for Pse5Ac7Ac and Pse5Ac7Am synthesis and transfer.

### Strain-Level Differences in FlaGrab Binding Correlate With Differences in PseD Sequence

We next examined whether amino acid sequence differences in PseA or PseD encoded by 12661 or 12664 could explain the observed differences in FlaGrab binding to these strains, since these proteins are the only two genes known to be required for Pse5Ac7Am display on flagella (the known FlaGrab receptor) in 11168 that are not required for flagellar filament biogenesis (Guerry et al., 2006). At the protein level, we found that PseA was upwards of 98.7% identical among strains, but that PseD diverged among strains (Figure S4A). PseD sequences from 11168, 12567, and 12660 were upwards of 96.9% identical to one another, but sequences from 12661 and 12664 were only 79.6–83.4% identical to PseD from 11168, 12567, and 12660. PseD sequences from 12661 and 12664 were 93.6% identical to one another. Most of the sequence divergence occurred within the third quarter of the PseD amino acid sequence. The fact that the two strains observed to express the most substantial reduction in FlaGrab binding grouped apart from the other strains based on their PseD sequence supported the hypothesis that changes in PseD sequence might explain the reduced FlaGrab binding of strains 12661 and 12664.

### *C. jejuni* 12661 Encodes a 25-Nucleotide Insertion Containing a Poly-G Tract in *pseD*

**Figure 5:**
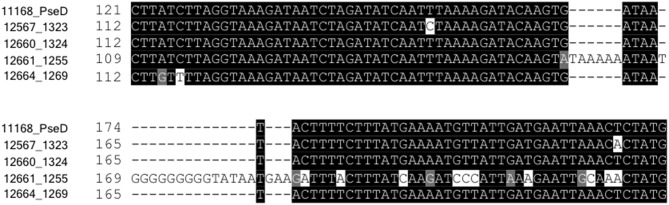
Strain 12661 harbors a poly-G tract-containing insertion in *pseD* that is not present in strains 11168, 12567, 12660, or 12664. Nucleotide sequence alignment of an internal region of *pseD* for all strains showing the 25 inserted nucleotides in strain 12661, nine of which make up a poly-G tract.

The fact that FlaGrab binding to 12661 flagella occurred variably, while 12664 displayed a consistent lack of FlaGrab binding, pointed to the existence of differences in Pse5Ac7Am expression between these strains. This prompted us to more closely examine the nucleotide sequence identity of *pseD* from these two strains, which led us to discover a 25-nt insertion near the 5′ end of *pseD* in 12661 that is not present in any of the other strains (Figure 5). Interestingly, this insertion included a 9-G homopolymeric (poly-G) tract. Homopolymeric tracts are commonly signs of phase-variability in bacterial genes, and have been implicated in *C. jejuni* phage-host interactions (Coward et al., 2006; Sørensen et al., 2012). We analyzed the raw Illumina MiSeq reads from our whole genome sequence analysis of 12661 (Sacher et al., 2018b), and indeed found variability in the number of Gs at this locus, supporting the notion that this strain could encode a phase-variable version of *pseD* (data not shown).

### FlaGrab Pressure Does Not Select for *pseD-*Off Variants in 12661 Under Our Conditions

To test the hypothesis that slipped-strand mispairing in *pseD* drives variation in 12661 binding to FlaGrab, we first amplified the *pseD* region from five single colony isolates, then from a mixed population of cells, and then from purified genomic DNA, all in the absence of FlaGrab, and assessed poly-G tract lengths using Sanger sequencing. To ensure the region we were amplifying was *pseD* and not a homologous sequence, since *C. jejuni* strains tend to encode 6–7 highly similar *maf* (motility associated factor, including *pseD*) genes at the flagellar glycosylation locus, we designed our primers to amplify a region anchored within *ptmA*, the gene directly upstream of *pseD*. We found that in all cases, the poly-G tract in *pseD*_12661 was in the “on” (9 Gs) state, suggesting in-frame expression of PseD (data not shown). We next sought to determine whether FlaGrab binding would provide a pressure for the strain to phase-vary its *pseD*. We grew 12661 in the presence of FlaGrab and amplified *pseD* from 12661 within and outside the spot of FlaGrab-induced clearing using colony PCR followed by Sanger sequencing. Sequence analysis of amplicons derived from FlaGrab-exposed cells (*n* = 2 sections) and FlaGrab-unexposed cells (*n* = 3 sections) showed that the poly-G tract remained phased-on in each case, with no apparent secondary peaks underneath the primary nucleotide identified at each location (data not shown). From this, we concluded that under the conditions tested, FlaGrab pressure does not select for off-switching of *pseD* in *C. jejuni* 12661.

### *C. jejuni* Strains 12661 and 12664 Display Flagellar Glycan Profiles Distinct From One Another and From 11168

Along with the differences in *pseD* sequence observed in 12661 and 12664, we wanted to determine whether these strains, which displayed reduced FlaGrab binding, displayed flagellar glycans distinct from 11168, which displays robust FlaGrab binding. To test this, we used high-resolution liquid chromatography with tandem mass spectrometry (LC-MS/MS) to identify and compare the glycans present on trypsin-digested flagella isolated from 12661 (in its non-FlaGrab-binding state), 12664 and 11168. We hypothesized that we would see reduced levels (or a complete absence) of Pse5Ac7Am on 12661 and 12664 flagella compared with 11168, and we surmised that other glycans would instead be present on the flagella of 12661 and 12664.

**Figure 6:**
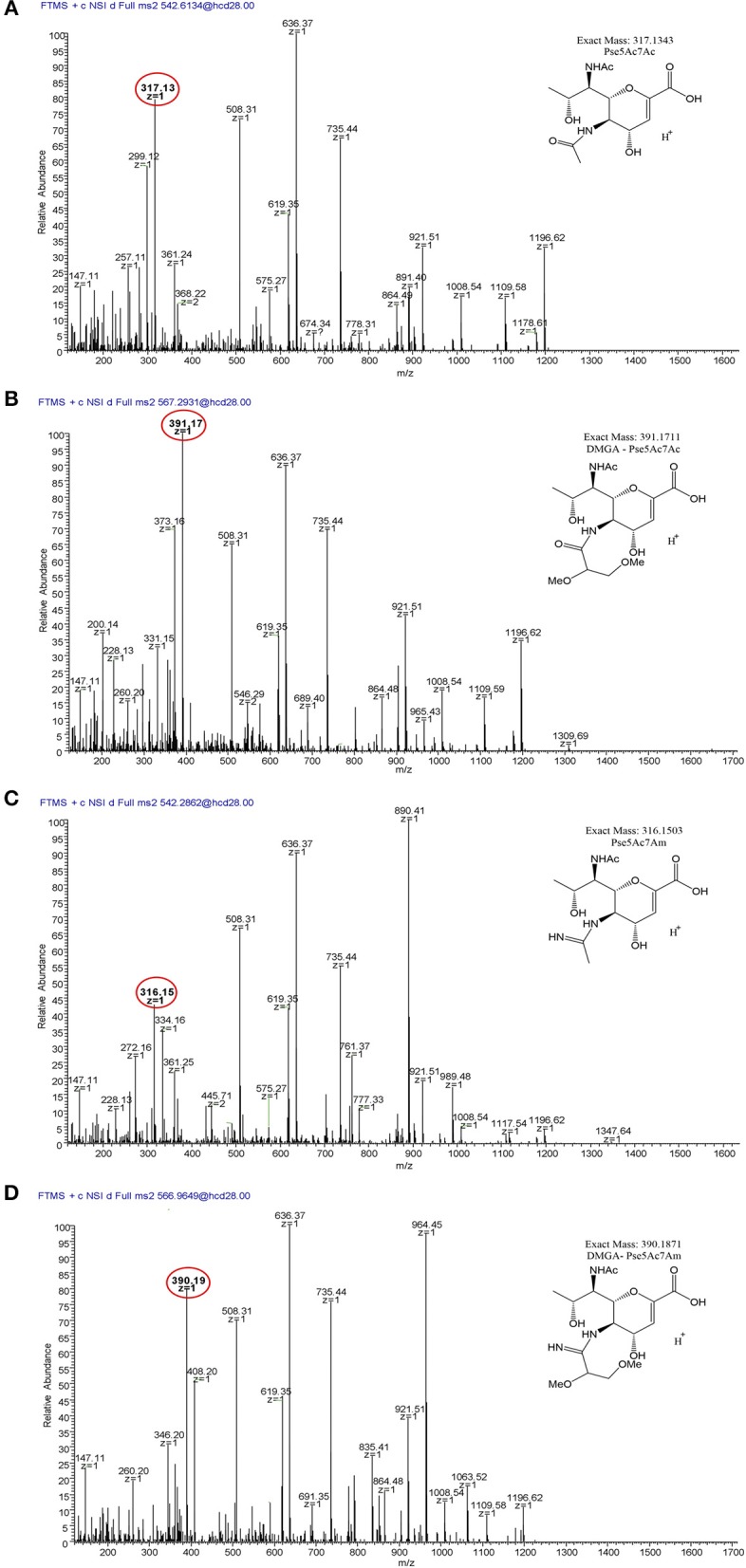
LC/MS-MS fragmentation spectra of trypsin-digested *C. jejuni* 11168 flagellar glycopeptide ^180^ISTSGEVQFTLK^191^. **(A)** ^180^ISTSGEVQFTLK^191^ + Pse5Ac7Ac (oxonium ion m/z = 317.13); **(B)** ^180^ISTSGEVQFTLK^191^ + Pse5Ac7Am (oxonium ion m/z = 316.15); **(C)**, ^180^ISTSGEVQFTLK^191^ + DMGA-Pse5Ac7Ac (oxonium ion m/z = 391.17); **(D)** ^180^ISTSGEVQFTLK^191^ + DMGA-Pse5Ac7Am (oxonium ion m/z=390.19). Oxonium ions are circled, and proposed structures of monosaccharides are shown.

**Figure 7:**
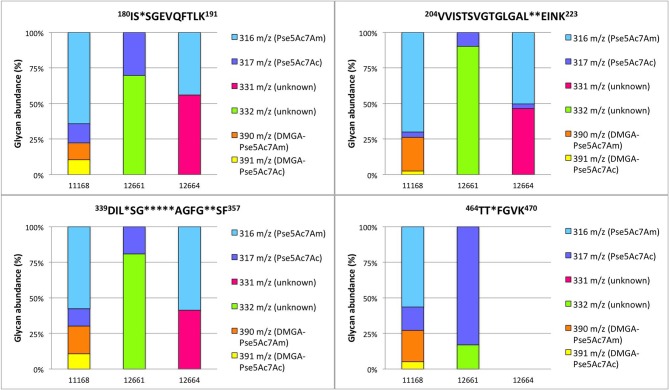
Percent relative abundance of flagellar glycans presented by *C. jejuni* 11168, 12661, and 12664 for four detected *C. jejuni* FlaA/FlaB peptides as determined by LC-MS/MS. In 12664, the peptide ^464^TTAFGVK^470^ was not detected, and thus no bar is depicted.

To verify our methodology, we first examined 11168 flagellar glycans. In agreement with Ulasi et al. (2015), we detected four glycopeptides: ^180^ISTSGEVQFTLK^191^, ^204^VVISTSVGTGLGALADEINK^223^, ^339^DILISGSNLSSAGFGATQF^357^, and ^464^TTAFGVK^470^, and identified several glycoforms for each (Figures 6, 7) (Ulasi et al., 2015). Also in agreement with Ulasi et al. and others, we detected oxonium ions corresponding to Pse5Ac7Ac (m/z = 317.13), Pse5Ac7Am (m/z = 316.15), dimethylglyceric acid (DMGA)-Pse5Ac7Ac (m/z = 391.16), and DMGA-Pse5Ac7Am (m/z = 390.18) (Logan et al., 2009; Zampronio et al., 2011; Ulasi et al., 2015). We also compared the relative glycan abundances at each location by comparing the relative peak intensities between the analyzed glycopeptides. We observed that for all four peptides, 3.81–16.44% of the glycan content corresponded to Pse5Ac7Ac, while 56.39–69.89% corresponded to Pse5Ac7Am or its Leg derivative, 2.64–10.41% corresponded to DMGA-Pse5Ac7Ac, and 12.01–23.65% corresponded to DMGA-Pse5Ac7Am (Figure 7).

**Figure 8:**
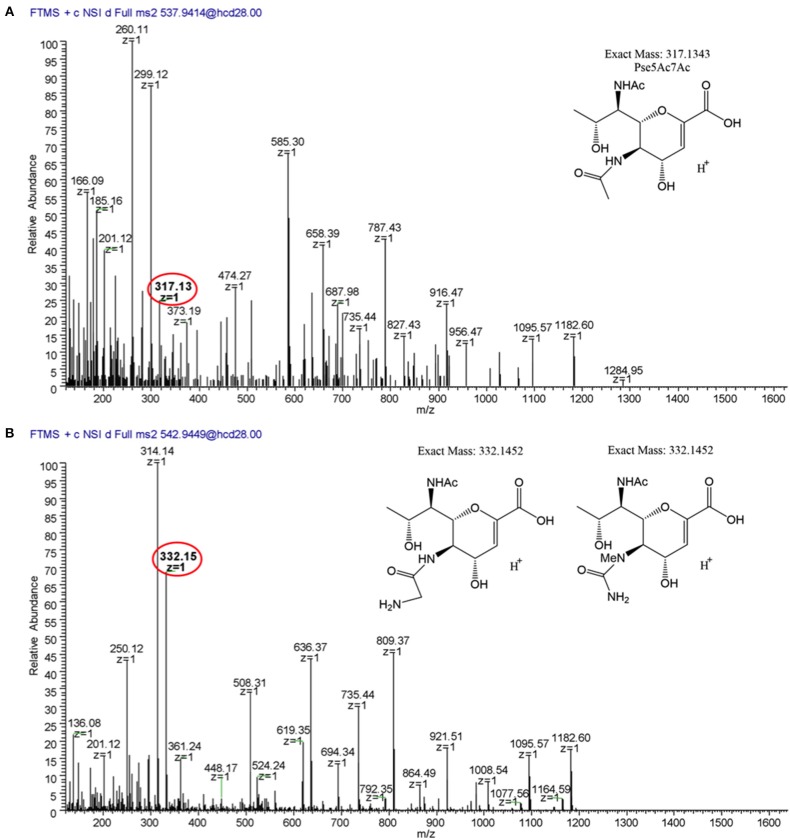
LC/MS-MS fragmentation spectra of trypsin-digested *C. jejuni* 12661 flagellar glycopeptide ^180^ISSSGEVQFTLK^191^. **(A)** MS^2^ spectrum of ^180^ISSSGEVQFTLK^191^ + a glycan of oxonium ion m/z = 317.13 (C_13_H_21_O_7_${\text{N}}_{2}^{+}$), **(B)** MS^2^ spectrum of ^180^ISSSGEVQFTLK^191^ + a glycan of oxonium ion m/z = 332.15 (C_13_H_22_O_7_${\text{N}}_{3}^{+}$). Oxonium ions are circled, and proposed structures of monosaccharides are shown.

We next sought to analyze 12661 flagellar peptides. To confirm the non-FlaGrab-binding state of the 12661 population used for MS, we performed FlaGrab immunogold labeling/TEM on the same cell preparation as used to prepare flagella for MS, and confirmed that the protein did not bind to flagella in that preparation (data not shown). Our MS analysis of trypsin-digested 12661 flagellin resulted in the detection of the four glycopeptides described for 11168, ^180^ISTSGEVQFTLK^191^, ^204^VVISTSVGTGLGALADEINK^223^, ^339^DILISGSNLSSAGFGATQF^357^, and ^464^TTAFGVK^470^ (Figure 8). On all four peptides, we detected oxonium ions of m/z = 317.13 (Figure 8A), which corresponds to Pse5Ac7Ac. Also on all four peptides, we detected an oxonium ion of m/z = 332.15 (Figure 8B), which does not correspond to any known *Campylobacter* flagellar glycan, but based on the addition of 15 Da to the mass of Pse5Ac7Ac, may represent the addition of an amine group to this glycan (the proposed structure is depicted in Figure 8B, inset). Notably, we did not detect oxonium ions corresponding to Pse5Ac7Am, DMGA-Pse5Ac7Ac or DMGA-Pse5Ac7Am. In terms of relative glycan content, we observed that for the first three peptides, glycans corresponding to Pse5Ac7Ac made up 9.78–30.31% of the glycan content, while the remaining 69.69–90.22% of the glycan content corresponded to the unknown glycan of m/z = 332.15 (Figure 7). However, the ^464^TTTFGVK^470^ peptide displayed the opposite trend, with 83.00% of the glycan content corresponding to Pse5Ac7Ac and 17.00% corresponding to the unknown glycan of m/z = 332.15.

**Figure 9:**
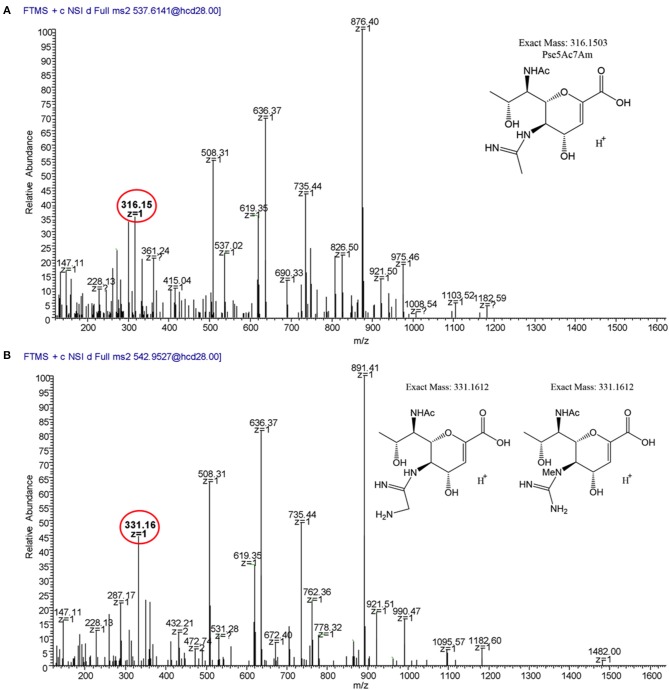
MS^2^ fragmentation spectra of trypsin-digested *C. jejuni* 12664 flagellar glycopeptide ^180^ISSSGEVQFTLK^191^. **(A)** MS^2^ spectrum of ^180^ISSSGEVQFTLK^191^ + a glycan of oxonium ion m/z = 316.15 (C_13_H_22_O_6_${\text{N}}_{3}^{+}$); **(B)** MS^2^ spectrum of ^180^ISSSGEVQFTLK^191^ + a glycan of oxonium ion m/z = 331.16 (C_13_H_23_O_6_${\text{N}}_{4}^{+}$). Oxonium ions are circled, and proposed structures of monosaccharides are shown.

We next analyzed 12664 flagellin. We detected the first three glycopeptides described above, but not the ^464^TTAFGVK^470^ peptide, so its glycan profile could not be determined (Figures 7, 9). Interestingly, upon analysis of the three glycopeptides detected, we detected oxonium ions of m/z = 316.15, which corresponds to Pse5Ac7Am. Also on all three glycopeptides, we detected an unknown glycan of m/z = 331.16. Based on the addition of 15 Da to the mass of Pse5Ac7Am, this may represent the addition of an amine group to this glycan (the proposed structure is depicted in Figure 9B, inset). In addition, in this strain, as for 12661 but not for 11168, we did not detect the DMGA derivatives DMGA-Pse5Ac7Ac or DMGA-Pse5Ac7Am. In terms of glycan content, the glycan corresponding to Pse5Ac7Am was 43.86–58.60% abundant across the three detected glycopeptides, while the unknown glycan of m/z = 331 was 41.40–56.14% abundant. Also, the glycan corresponding to Pse5Ac7Ac was 3.17% abundant on the ^204^VVISTSVGTGLGALVEEINK^223^ peptide only, but was not detected on the others.

### FlaGrab Binding to *C. jejuni* 11168 Flagellar Glycans Does Not Require Dimethylglyceric Acid (DMGA)

**Figure 10:**
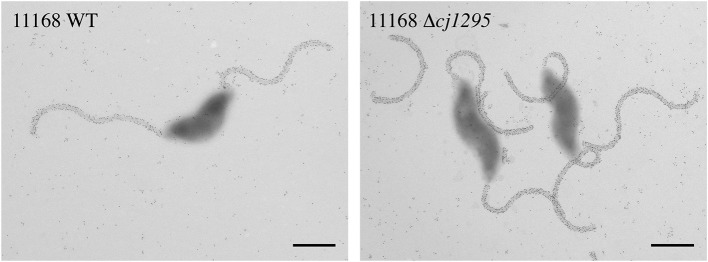
Transmission electron micrographs of FlaGrab-labeled *C. jejuni* 11168 and 11168Δ*cj1295*. Images are representative of experiments done in duplicate. Scale bars represent 800 nm.

To rule out the possibility that the absence of DMGA-Pse5Ac7Am was the reason for the lack of FlaGrab binding in 12664 and 12661, we sought to determine whether FlaGrab binding to Pse5Ac7Am requires DMGA in 11168. Hitchen et al. (2010) have shown that a *cj1295* mutant in *C. jejuni* 11168 is unable to synthesize DMGA-Pse5Ac7Ac or DMGA-Pse5Ac7Am (Hitchen et al., 2010). We therefore generated a *cj1295* mutant in 11168 and used immunogold labeling/TEM to test whether FlaGrab binding still occurred in this strain. We found no difference in FlaGrab binding between the *cj1295* mutant and wild type cells (Figure 10), suggesting that DMGA is not required for FlaGrab binding to Pse5Ac7Am.

### Differences in 11168, 12661, and 12664 Gene Content at the Flagellar Glycosylation Locus Are Primarily Among *maf* and DUF2920-Containing Genes

To gain insight into the genetic basis for the observed differences in flagellar glycans detected on 11168, 12661, and 12664 flagella, we examined the differences in gene content at the flagellar glycosylation locus of each strain. In particular, we hypothesized that 12661 and 12664 would encode genes in this locus not encoded by 11168 that might explain their display of unknown glycans of m/z = 332 and 331 glycans, respectively. Notably, the m/z value for each unknown glycan represents a gain of ~15 Da relative to the other glycan detected for that strain (m/z = 317 and 316, respectively), suggesting that an amine group (NH_2_, 16 Da) might substitute for a hydrogen atom on the glycan detected in each case. We therefore examined the genes within the flagellar glycosylation locus that were only present in 12661 and 12664 to see if any had homology to an aminotransferase, but did not identify any aminotransferase homologs.

To identify candidate genes that might be involved in the synthesis of the unknown glycans of m/z = 332 and 331, we examined other differences in gene content at the flagellar glycosylation locus between 12664, 12661, and 11168. We noted strain-specific differences in *maf* gene content and in distribution of poly-G tracts among these genes. Differences in *maf* genes were particularly noteworthy, as paralogues of these (*pseD, pseE*) are known to be involved in pseudaminic acid transfer to flagellin in *Campylobacter*. For instance, strain 12664 lacks the motility associated factor *maf5* (*cj1341c*), while *maf6* (*cj1342c*) is missing from both 12664 and 12661 (Figure 4, Region I). As well, the poly-G tract in *maf2* is uniquely absent in 12661. We also noted differences in the number, location, and presence/absence of poly-G tracts in genes containing a DUF2920 domain (DUF = domain of unknown function) (Figure 4, Regions C, D, and I). In 12664, we noted the unique presence of the DUF2920-containing gene *CJ12664_1248* downstream of the identically-annotated *cj1310c* (Figure 4, Region D). In addition, we found that 12664 encodes another DUF2920-containing gene, *CJ12664_1277*, just upstream of *maf4* (*cj1340c*) (Figure 4, Region I), and we found that 12661 also encoded this second DUF2920-containing gene (*CJ12661_1263)*. However, strain 12661 is also missing the DUF2920-containing gene *cj1306* (Figure 4, Region C). While strains 11168 and 12664 encode a poly-G tract in *cj1305c*, another DUF2920-containing gene, this poly-G tract is disrupted in strain 12661. Outside of *maf* and DUF2920-containing genes, strain 12664 also uniquely lacks the *fkbH* domain-containing gene *cj1302* (Figure 4, Region B) (it is present as a pseudogene), and 12661 uniquely lacks *cj1301*, which is annotated as an epimerase (Figure 4, Region B).

## Discussion

In this study, we sought to understand how FlaGrab, a protein encoded by all *C. jejuni* phages characterized to date (Javed et al., 2014), inhibits the growth of *C. jejuni* 11168 by binding to its flagella, and to understand how some strains of *C. jejuni* are able to resist this effect (Sacher, 2018). Ultimately, our goal was to gain insight into how phage pressures might contribute to *C. jejuni* flagellar glycan diversity and complexity by studying susceptibility and resistance to a phage protein with both an inhibitory effect on cells and a specificity for certain surface-exposed glycans.

First, we examined the growth inhibitory effect that resulted from FlaGrab binding to *C. jejuni* flagella. Other organisms, such as *Bacillus subtilis*, have been shown to alter biofilm-related gene expression in response to flagellar contact with a surface (which could be recapitulated through binding by flagella-specific antibodies) (Cairns et al., 2013; Belas, 2014). Therefore, we hypothesized that FlaGrab binding to *C. jejuni* flagella might inhibit flagellar rotation, which might in turn send a signal through the flagella that would alter gene expression in a way that would reduce cellular growth rate. As *C. jejuni* has previously been shown to alter its protein expression during biofilm association (Kalmokoff et al., 2006), we surmised that it might have evolved a mechanism of sensing and responding to surface contact, and that FlaGrab binding might be stimulating such a pathway. To test whether *C. jejuni* gene expression was altered in response to FlaGrab binding, we analyzed total gene expression changes in cells treated with the C-terminal (binding) component of FlaGrab, CC-FlaGrab, by RNA-seq. We found that genes involved in the TCA cycle, oxidative phosphorylation, and carbon metabolism were downregulated upon FlaGrab treatment, reflecting an overall metabolic downshift that is not surprising in light of the reduced growth observed. Interestingly, the pattern of FlaGrab-induced altered gene expression we have observed in *sdhA* (*mrfA*) and *sdhB* (*mrfB*) is mirrored by the gene expression changes observed by Guccione et al. (2017) in response to aerobic growth of *C. jejuni* (Guccione et al., 2017). One can speculate that FlaGrab may induce a mild form of oxidative stress on exposed cells. In support of this notion, we previously observed that *C. jejuni* 11168 upregulates expression of oxidative stress response genes in response to infection by phage NCTC 12673, the phage from which the FlaGrab protein described here originates (Sacher et al., 2018c). Further work into understanding the connection between phage infection, oxidative stress and oxidative stress response in *C. jejuni* is merited.

We next sought to examine the mechanism by which FlaGrab might lead to the observed effects on cells. *C. jejuni* flagellar rotation is hypothesized to be driven by proton motive force through the MotA and MotB stator proteins, as in many other motile bacteria (Chaban et al., 2018), and altered proton flow has been shown to increase flagellar torque in other bacteria (Lele et al., 2013). In addition, *C. jejuni* cells are known to modulate their swimming speed according to viscosity (Szymanski et al., 1995). We therefore hypothesized that FlaGrab binding, which occurs all along the length of the flagella of susceptible *C. jejuni* cells, might lead cells to sense increased drag, which might induce the flagellar motor complex to increase proton flow through the motor channel to compensate. This increased proton flow would presumably disrupt proton motive force homeostasis in the cell, perhaps due to electron leakage across the electron transport chain, as has been proposed by Flint et al., which could explain the observed growth inhibition (Flint et al., 2014). To begin to examine this hypothesis, we tested whether FlaGrab could inhibit the growth of *motA* or *motB* mutants, which have a “paralyzed” flagellar phenotype as they each lack a key element of the flagellar motor (Ribardo et al., 2019). We predicted that these mutants would be bound, but not cleared by FlaGrab. Consistent with our prediction, we found that *motA* and *motB* mutants were both completely resistant to FlaGrab growth inhibition, and yet FlaGrab binding was normal, suggesting that clearance activity does indeed require flagellar motor function. Further work is required to demonstrate that FlaGrab binding to functional flagella indeed alters proton flow, and to determine whether this disruption is sufficient to explain the observed growth inhibition. Still, our results are consistent with a model whereby FlaGrab binding to flagella is “mechanosensed” by the cell through detection of increased drag on rotating flagella.

Next, we wanted to understand the mechanisms by which *C. jejuni* might resist FlaGrab activity. To do this, we identified two *C. jejuni* strains with reduced susceptibility to FlaGrab-induced growth inhibition, 12661 and 12664. We first sought to determine whether a lack of motility might explain their resistance. Strain 12664 displayed similar motility levels to 11168, suggesting that a lack of motility was not the reason for its FlaGrab resistance. However, 12661 was 49% less motile, which could explain part of this strain's reduced susceptibility to FlaGrab activity. We next sought to examine the levels of FlaGrab binding to 12664 and 12661. We determined that both strains exhibited reduced FlaGrab binding, suggesting that FlaGrab resistance may be a consequence of reduced expression of the FlaGrab receptor (Pse5Ac7Am). Interestingly, strain 12661 was variably susceptible to both FlaGrab-induced growth inhibition and binding, while strain 12664 was consistently FlaGrab-resistant. These results suggested that each strain had evolved to avoid FlaGrab binding in its own way, and suggested that 12661 might encode a phase-variable version of a gene required for FlaGrab receptor expression.

We next wanted to further elucidate how *C. jejuni* 12661 and 12664 each escaped FlaGrab binding. We hypothesized that these strains might avoid FlaGrab binding either by not encoding Pse5Ac7Am at all, by encoding mechanisms of downregulating or off-switching their Pse5Ac7Am expression, or by expressing other glycans able to render Pse5Ac7Am unrecognizable to FlaGrab. We first compared the genes each strain encoded at the flagellar glycosylation locus with 11168, and found that both 12661 and 12664 encode full-length copies of all genes known to be required for Pse5Ac7Am synthesis and display on flagella (Logan et al., 2008). However, we found that strains 12661 and 12664 clustered separately from FlaGrab-binding strains, such as 11168, according to their PseD sequence (Figure S4). PseD is considered to be responsible for Pse5Ac7Am transfer onto *C. jejuni* flagellin, as Pse5Ac7Am accumulated in the cytosol but was not detected on the flagella of a *pseD* mutant in *C. jejuni* strain 81–176 (Guerry et al., 2006; McNally et al., 2006). It is plausible that the observed sequence differences in PseD in strains 12661 and 12664 might lead to changes in the ability of PseD to transfer Pse5Ac7Am that could explain the reduced FlaGrab binding to these two strains. Further work into understanding the active site of PseD, as well as the predicted effects of the observed sequence differences on its function, are merited. Interestingly, *pseD* polymorphisms have recently been implicated in *C. jejuni* persistence in humans: in a study by Crofts et al., a single *C. jejuni* strain was used to infect several volunteers, and whole genome sequencing of isolates from subjects who experienced severe and/or recurrent infections revealed an enrichment of *pseD* gene variants (Crofts et al., 2018). Further understanding of PseD function and the factors contributing to selection of variants, particularly as they relate to phage-host interactions (which are understudied in mammalian systems), may thus yield important data, and should be pursued in the future.

We next set out to explain why strain 12661 was variably bound by FlaGrab. The simplest explanation was phase variation at either *pseA* or *pseD*, as these are the two *C. jejuni* genes known to be involved in Pse5Ac7Am display that can be disrupted without abolishing flagellar filament biogenesis (Guerry et al., 2006). Phase variation is common in *C. jejuni*, and has been implicated in *C. jejuni* phage-host interactions (Coward et al., 2006; Sørensen et al., 2012). Interestingly, we found that 12661 expressed a poly-G-tract-containing version of *pseD*, while the other strains did not. However, we did not observe phase variation at this locus, even when cells were exposed to FlaGrab. Therefore, we concluded that variation in *pseD* was not the explanation for the variable FlaGrab binding phenotype we observed.

To determine whether FlaGrab-resistant strains expressed less Pse5Ac7Am, we analyzed the flagellar glycans of these strains compared to 11168. We found that the oxonium ion corresponding to Pse5Ac7Am, the known receptor for FlaGrab, was not identified on 12661 flagella, suggesting that this strain's resistance to FlaGrab was indeed mediated by the loss of this glycan. Conversely, flagella from strain 12664 did produce an oxonium ion with a mass to charge ratio consistent with Pse5Ac7Am. It is likely that this glycan corresponds not to Pse5Ac7Am, but instead to Leg5Am7Ac, a glycan of the same mass known to be displayed on *C. coli* VC167 flagella (McNally et al., 2007), which we have shown to be insufficient for FlaGrab binding (Javed et al., 2015a). In support of this explanation, strain 12664 encodes the genes (*cj1321–1325/6*) previously shown by Howard et al. (2009) to be involved in Leg5Am7Ac synthesis (Howard et al., 2009), while strain 12661 does not. Unfortunately, our efforts to obtain sufficient quantities of cytoplasmic nucleotide-activated sugars from 12664 to conclusively identify the glycan using NMR have thus far been unsuccessful. Of note, if 12664 does express Leg5Am7Ac and not Pse5Ac7Am, it would still call to question why Pse5Ac7Am is not also present. Although *C. coli* VC167 has been observed only to express Leg5Am7Ac and not Pse5Ac7Am, this strain only encodes the genes for the former (McNally et al., 2007), while strain 12664 encodes all genes required for both glycans (McNally et al., 2007). If both glycans are present on 12664 flagella, an alternate explanation for the lack of FlaGrab binding could be that the unknown glycan of m/z = 331 shields the FlaGrab binding epitope. Further work into understanding the mechanism behind the lack of FlaGrab binding in this strain is on-going.

As both 12661 and 12664 flagella lacked any oxonium ions corresponding to the DMGA-containing sugars found on 11168 flagella, we wanted to rule out the possibility that DMGA-Pse5Ac7Am was the required glycan for FlaGrab binding. We mutagenized *cj1295* in *C. jejuni* 11168, as this gene was shown to be responsible for DMGA-Pse5Ac7Ac and DMGA-Pse5Ac7Am synthesis in this strain (Hitchen et al., 2010). Since we observed no difference in FlaGrab binding to 11168 compared to the *cj1295* mutant, we concluded that DMGA is not part of the receptor required for FlaGrab binding to flagella.

During this study, we identified two novel glycan masses expressed on *C. jejuni* flagella: oxonium ions of m/z = 332 and 331. As these m/z ratios are equal to 317 + 15 and 316 + 15, we propose that they correspond to Pse5Ac7Ac and Pse5Ac7Am (or their legionaminic acid equivalents) with the addition of an amine group (NH_2_, 16 Da) in place of a hydrogen atom. We sought to identify candidate genes within the flagellar glycosylation loci that might be involved in the synthesis of the novel glycans we identified. Compared with 11168, the main differences we observed were in number and location of *maf* and DUF2920-containing genes. Phenotypes have been identified for some of the *maf* genes in *C. jejuni*, including *pseD, pseE*, and *maf4* (involved in Pse5Ac7Am transfer to flagellin, Pse5Ac7Ac transfer to flagellin, and CO_2_ or C_2_H_2_O_2_ transfer to Pse5Ac7Ac, respectively), but the role of most *maf* genes is unknown (Karlyshev et al., 2002; van Alphen et al., 2008). As for DUF2920 genes, even less is known about their function (Guerry et al., 2006). While it is possible that strain-specific *maf* and DUF2920 gene content could be responsible for the diversity in flagellar glycan expression we have uncovered here, it will undoubtedly be challenging to parse the roles of each in *C. jejuni* flagellar glycosylation. Nonetheless, our results point to these gene families as attractive targets for future studies.

## Conclusions

We have gained new insights into the mechanism of *C. jejuni* growth clearance by the conserved phage-encoded protein FlaGrab. Our results are consistent with a model whereby FlaGrab binds cells and transmits a mechanical signal through the flagella, which leads to slower cell growth. We also characterized the mechanism of FlaGrab resistance of two *C. jejuni* strains, and found that resistance was due to evasion of FlaGrab binding by altering flagellar glycan display through strain-specific mechanisms. We found that both strains display a different modified glycan not previously observed on *Campylobacter* flagella, and that the number and organization of *maf* and DUF2920-containing genes constitute the main differences at the flagellar glycosylation locus between FlaGrab-susceptible and -resistant strains. Together our results add to our understanding of the interactions between *C. jejuni* surface glycans and a conserved glycan-specific phage protein, and highlight unexplored diversity in *C. jejuni* flagellar glycosylation.

## Data Availability Statement

The datasets generated for this study can be found in the NCBI SRA archive #PRJNA580017.

## Author Contributions

JS designed and performed experiments, analyzed the data, and wrote the manuscript. ASh performed the mass spectrometry experiments and analyzed the data. RP performed microscopy and motility experiments. AF performed RNA sequencing. JB analyzed the RNA-Seq data. JB, ASt, PA, and DH provided materials and guidance, and analyzed the data. CS supervised and funded the project, analyzed the data, and edited the manuscript.

### Conflict of Interest

The authors declare that the research was conducted in the absence of any commercial or financial relationships that could be construed as a potential conflict of interest.
