# Supplementary material for: Binding of Phage-Encoded FlaGrab to Motile Campylobacter jejuni Flagella Inhibits Growth, Downregulates Energy Metabolism, and Requires Specific Flagellar Glycans
Source: Front Microbiol. 2020 Mar 20;11:397. doi: 10.3389/fmicb.2020.00397 (PMC7099621; doi:10.3389/fmicb.2020.00397)
Supplement: Supplementary file 1 [file Data_Sheet_1.docx]

# **Supplementary**


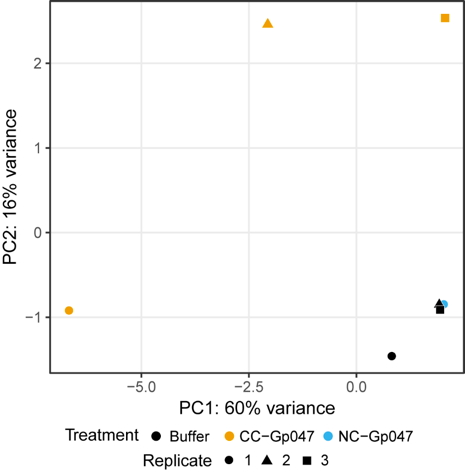


**Figure S1.** Principal component analysis of differentially-expressed *C. jejuni* 11168 genes 30 min following exposure to CC-FlaGrab, NC-FlaGrab or buffer. Plots were generated using the data from the 500 genes with the greatest variation in expression across samples.


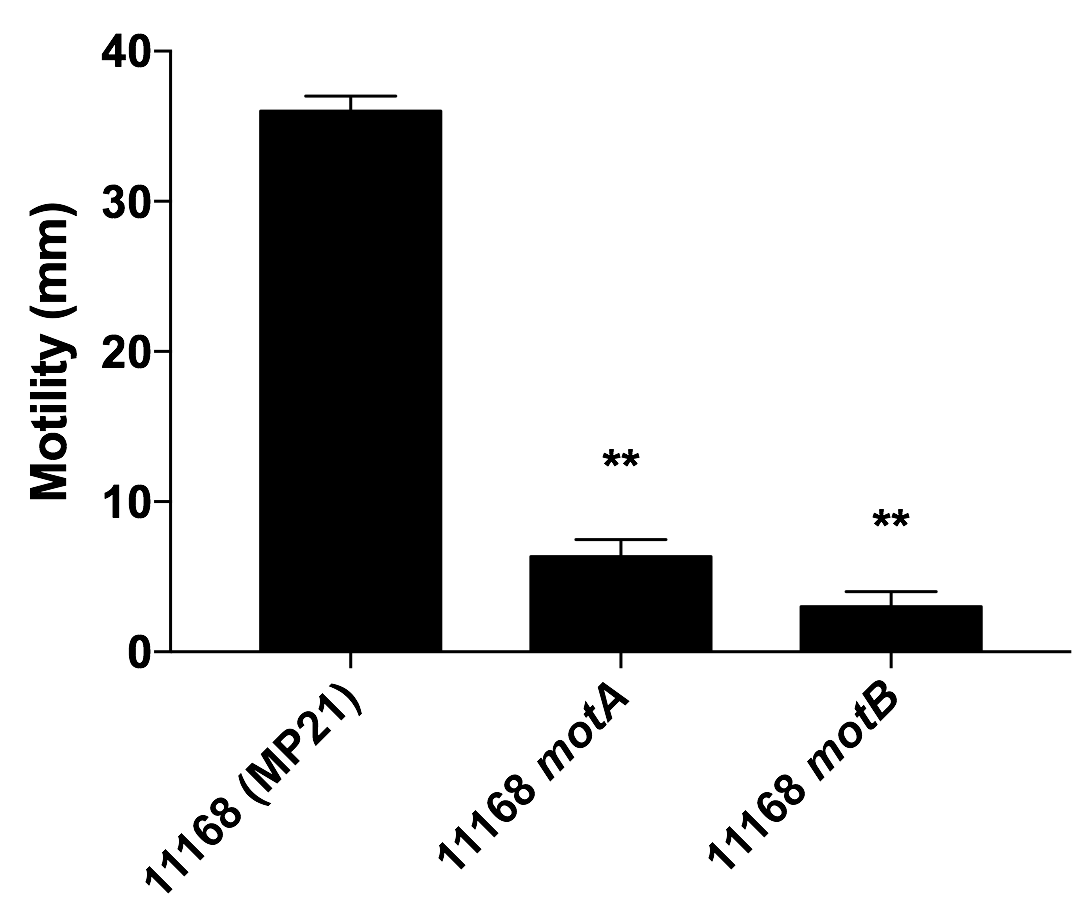


**Figure S2.** Motility of *Campylobacter jejuni* 11168 MP21 ∆*motA* and ∆*motB* mutants compared to wild type cells, as indicated by the diameter of cell spread in 0.4% MH agar after 47 h. Results depict the average, standard deviation and p-value (student’s T-test) for three replicates (p < 0.01 (**)).


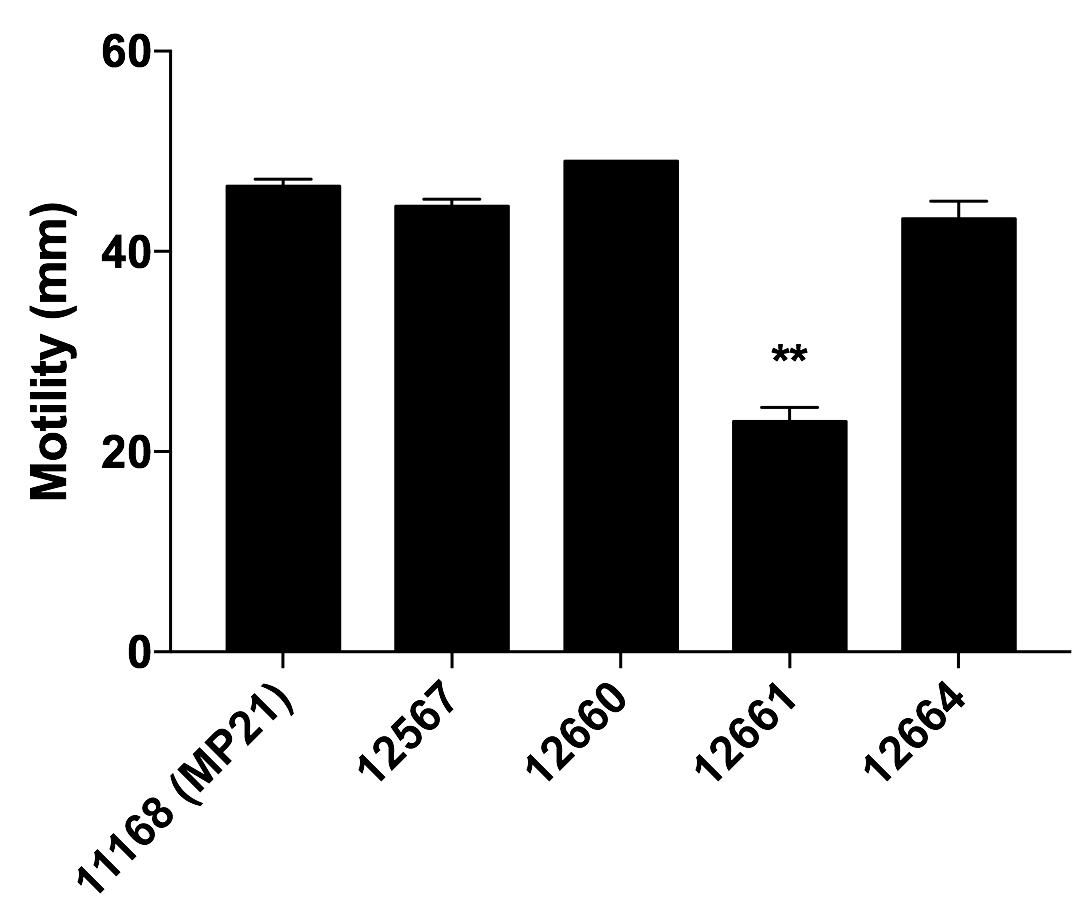


**Figure S3.** Motility of *Campylobacter jejuni* strains as indicated by the diameter of cell spread in 0.4% MH agar after 52 h. Results depict the average, standard deviation and p-value (student’s T-test) for two replicates (p < 0.01 (**)).

**A**

**
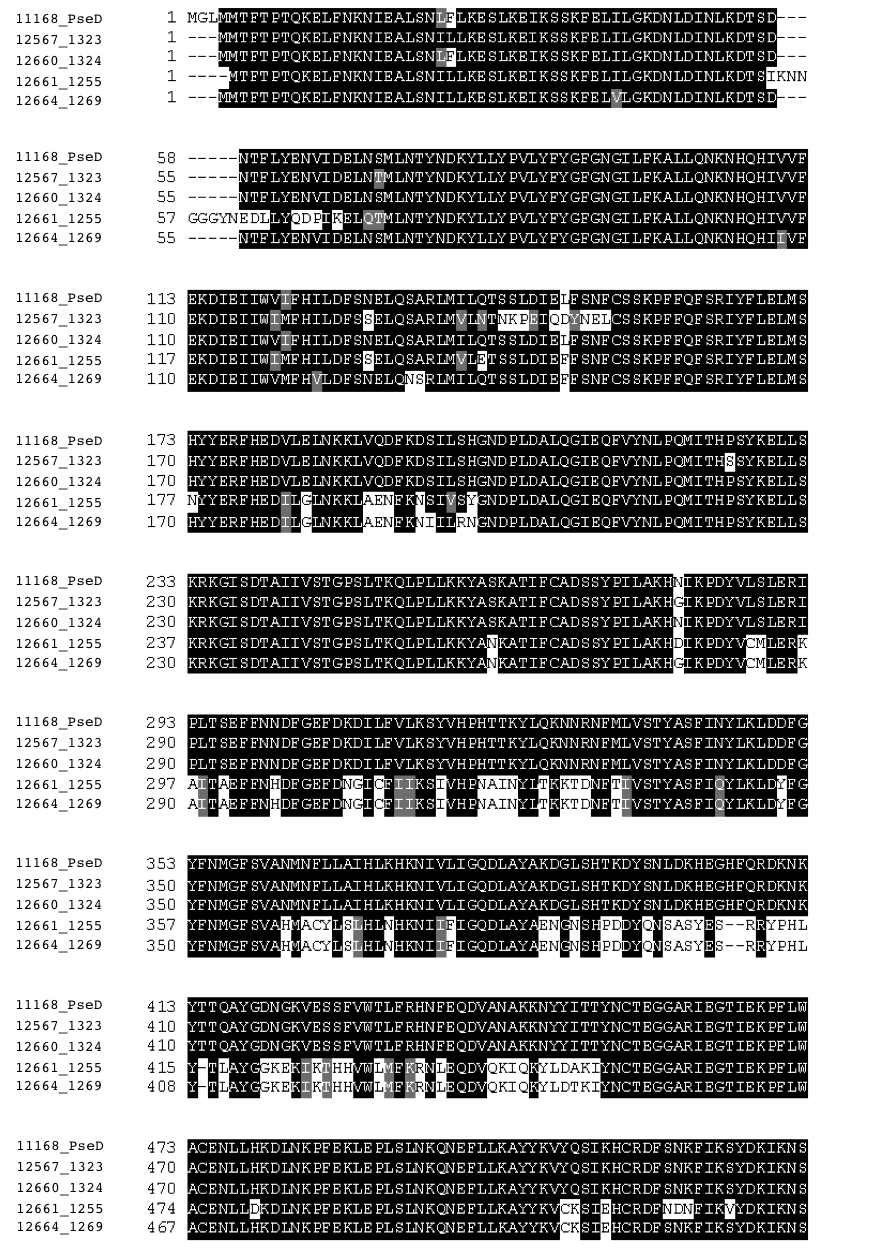
**

**B**

**
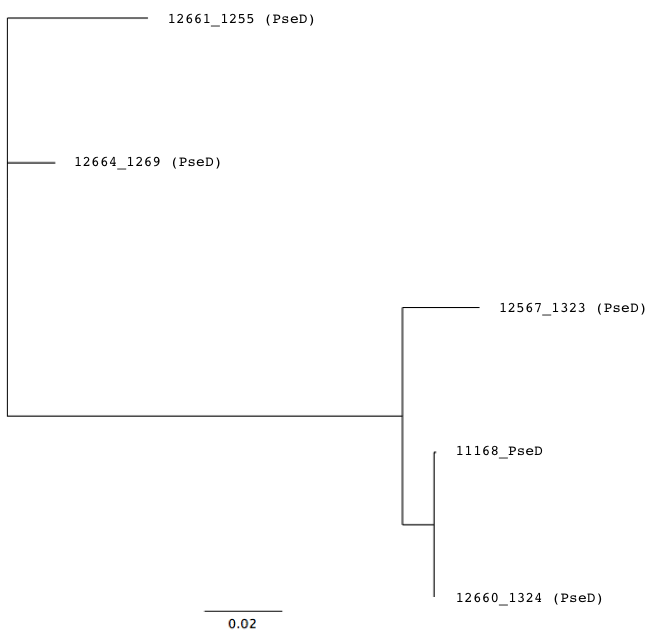
**

**Figure S4.** PseD amino acid sequence alignment (**A**) and a phylogenetic tree (**B**) depicting the relationship between PseD between the five strains.

**Supplementary Methods**

**Motility Assay**

Bacterial motility was tested in soft agar as described previously (Waseh *et al*., [2010](https://onlinelibrary.wiley.com/doi/full/10.1111/mmi.12849#mmi12849-bib-0046)). Briefly, Mueller Hinton plates containing 0.4% agar were prepared the day before the motility assay. After overnight growth (16-18 hrs), cells were suspended in PBS and set to an O.D_600_ of 1.0 in PBS. Five μL of the cell suspension was inoculated into the centre of each agar plate by lightly piercing the agar surface with a 10-μL pipette tip. Plates were allowed to dry undisturbed before being incubated right-side-up at 37^o^C overnight under microaerobic conditions suitable for *Campylobacter* growth. As an indicator of motility, the diameter of cell growth spread was measured with a ruler after 47-52 hours.

**References**

Waseh, S., Hanifi‐Moghaddam, P., Coleman, R., Masotti, M., Ryan, S., Foss, M., *et al*. (2010) Orally administered P22 phage tailspike protein reduces salmonella colonization in chickens: prospects of a novel therapy against bacterial infections. *PLoS ONE* 5: e13904.
